# Intravital Imaging Allows Organ-Specific Insights Into Immune Functions

**DOI:** 10.3389/fcell.2021.623906

**Published:** 2021-02-11

**Authors:** Selina K. Jorch, Carsten Deppermann

**Affiliations:** ^1^Institute of Experimental Immunology, University Hospital of Bonn, Rheinische Friedrich-Wilhelms Universität, Bonn, Germany; ^2^Institute of Clinical Chemistry and Laboratory Medicine, University Medical Center Hamburg-Eppendorf, Hamburg, Germany

**Keywords:** Intravital microscopy, liver, peritoneal cavity, lung, kidney, macrophage, neutrophil

## Abstract

Leukocytes are among the most mobile and versatile cells that have many essential functions in homeostasis and survival. Especially cells from the innate immune system, i.e., neutrophils and macrophages, play an important role as rapid first responders against invading microorganisms. With the advent of novel imaging techniques, new ways of visualizing innate immune cells have become available in recent years, thereby enabling more and more detailed discoveries about their nature, function and interaction partners. Besides intravital spinning-disc and 2-photon microscopy, clearing and 3D-imaging techniques provide new insights into the mechanism of innate immune cell behavior in their natural environment. This mini review focuses on the contributions of novel-imaging techniques to provide insight into the functions of neutrophils and macrophages under homeostasis and in infections. Imaging setups for different organs like the liver, kidney, heart, lung, and the peritoneal cavity are discussed as well as the current limitations of these imaging techniques.

## Introduction

The immune system is composed of many different cell types distributed throughout the body. While some immune cells are sessile, most are very motile, and able to migrate in tissues. Intravital microscopy had a huge impact on our understanding of the immune system as it is the only technique that allows to simultaneously study structure and function at a cellular to subcellular level with high spatial and temporal resolution *in vivo*.

The first attempt of looking into vessels to observe moving cells dates back more than a century ago (Pittet and Weissleder, 2011). Since then, imaging techniques have developed a lot and nowadays it is possible to visualize the immune processes taking place in almost every organ—brain, eye, lung, heart, lymph node, joints, spleen, liver, gut, kidney, bladder, peritoneal cavity, mammary ducts and more using experimental mouse, or rat models. The development of better and faster microscopy techniques had a tremendous impact. We moved from simple light microscopy to more complex techniques. From confocal microscopy including laser scanning (LSM) and spinning-disc (SDM), to multiphoton microscopy (MP). In confocal microscopes the light is focused on a single point and the emitted fluorescence goes thru a pinhole before reaching the detector, which results in less out-of-focus fluorescence. However, scanning the specimen point by point slows the process down significantly. A spinning-disc setup consists of multiple pinholes on a rotating disc which speeds up the scanning process significantly thereby allowing to capture fast events like bacteria or platelets moving in the blood stream in real time. However, the penetration depth of LSM and SDM are limited and in dense organs like the kidney, much better results are obtained with MP excitation. In 2-photon imaging, a pulsed-laser directs two exciting photons of about half the energy to the specimen. When these two low energy photons hit a fluorophore, they cause excitation to the same level as one high-energy photon, which contributes to a very specific focal point. Compared to single-photon imaging, no pinhole is necessary for a single focus point and in combination with the use of longer wavelengths this results in less out-of-focus excitation and reduced photodamage (Ntziachristos, 2010). The choice between using LSM/SDM or MP depends largely on the tissue and phenomenon of interest. In Table 1 we give an overview about the microscopy techniques mostly used for intravital imaging and their properties regarding speed, versatility, and imaging depth.

**Table 1:** Overview of microscopy techniques.

**Technique**	**Speed**	**Versatility**	**Intravital capability**	**Imaging depth**
Laser scanning	+	++	+	+
Confocal	+	++	+	+^**^
Spinning disk	+++	++	+++	+
Lightsheet	O	+	(+)^*^	+++
2-photon	++	+	++	++

* *Only in animals that are transparent, e.g., zebrafish embryos or pigment-deficient zebrafish*.

** *Higher penetration-depth of point scanners vs. spinning disk, e.g., observed in kidney*.

**Figure 1:**
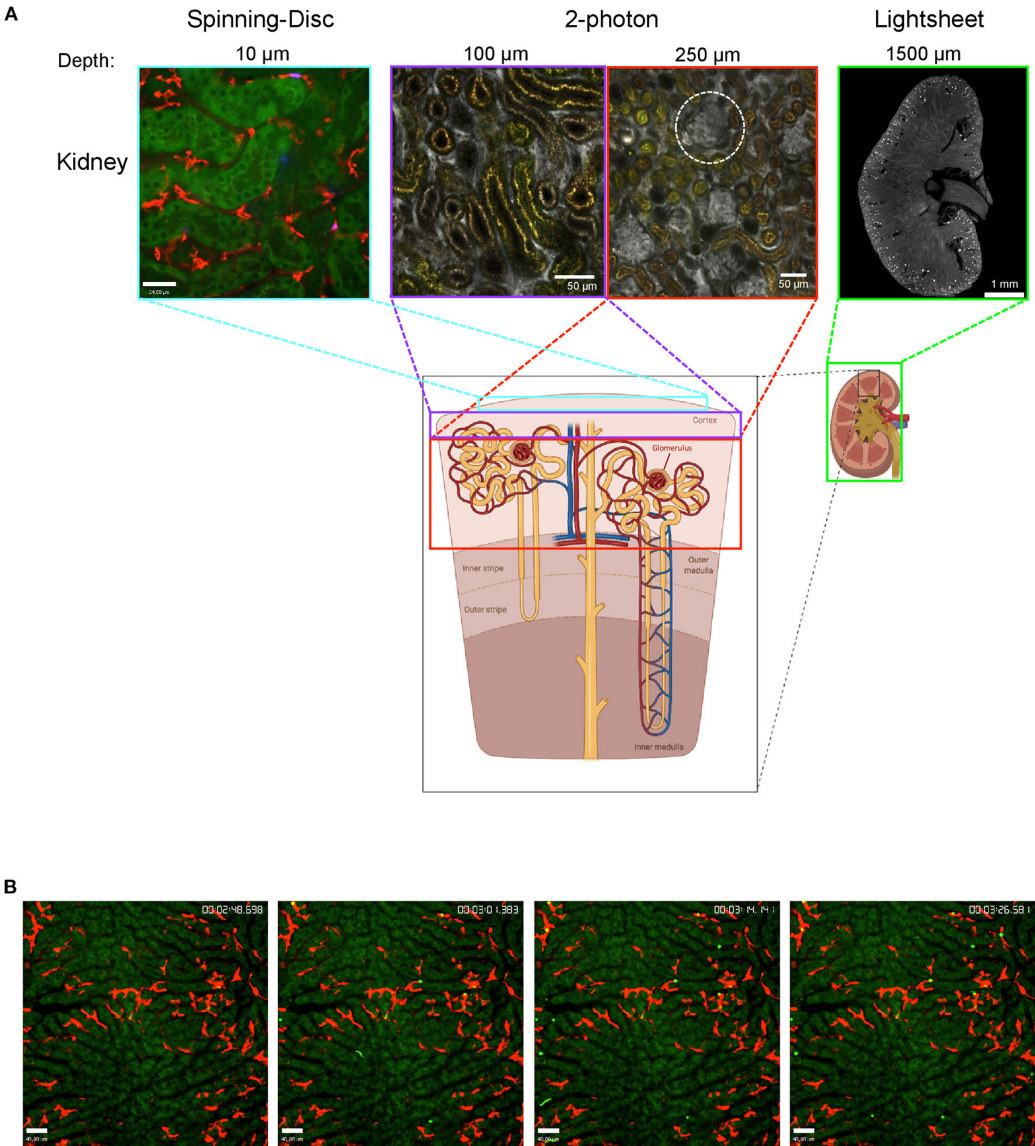
Imaging the mouse kidney and liver using different microscopy techniques. **(A)** Outer kidney cortex imaged using spinning-disc microscopy (left) showing autofluorescent tubules (green) and F4/80^+^ macrophages (red). Images obtained using 2-photon microscopy (center) at 100 and 250 μm tissue penetration showing vasculature (albumin, gray) with glomeruli (white-dotted circle) and autofluorescent tubulues (yellow). Lightsheet microscopy image showing a cross-section of the whole kidney (right) with vasculature stained (CD31, gray) and glomeruli (bright spots). The areas that can be visualized with each technique are indicated with colored boxes. Lightsheet image was provided by Dr. Alexander Böhner, Bonn. Sub-figure was created with BioRender.com. **(B)** Mouse liver was imaged using spinning-disc microscopy showing capture of bacteria (bright green) by Kupffer cells (F4/80, red) over time (timestamp top right); hepatocytes are autofluorescent (dark green).

Although intravital imaging is indispensable to gain insights into dynamic processes, it is important to know its limitations. The biggest is the imaging depth. Even with MP it is usually not possible to penetrate the whole tissue. The kidney is a very good example for this limitation. LSM/SDM is able to give beautiful details about the tubules in the outer layer of the cortex and new MP techniques allow to easily observe glomeruli which are located at the beginning of nephrons deep inside the kidney cortex, but until today it is almost impossible to image the medulla region with intravital techniques since it is located even further inside the dense kidney tissue. That is the point where clearing techniques can add valuable knowledge at least about the 3D spatial location of cells (Figure 1). Cleared organs can be analyzed with lightsheet microscopes to get an overview or with confocal techniques to image smaller structures at high resolution. Regardless of the microscopy techniques, another important point to always keep in mind is that the dark matter matters and can make up a big portion of the organs and cells we look at, as we can only see what is labeled or what is auto-fluorescent. In the following sections we summarize recent findings regarding neutrophils and macrophage behavior that could not have been achieved without intravital imaging or lightsheet microscopy.

### Visualizing the Rapid Capture of Bacteria Through Kupffer Cells in the Liver

Cellular interactions under dynamic conditions outside of solid tissues, e.g., in the bloodstream can be transient or permanent. Investigating this type of interactions using standard tissue histology can only provide a snapshot with no insight into the true nature of the interaction observed. Since the formation and dissolution of such interactions can be very fast, visualization requires high-imaging speed that can be provided by state-of-the-art spinning-disk intravital microscopy (SD-IVM) or microscopes using very fast (resonant) scanners.

One example of such an interaction which is rapidly formed and permanent is the removal of bacteria from the bloodstream. Using SD-IVM it was shown that sequestration of blood-borne staphylococci occurs by Kupffer cells, the tissue resident macrophages of the liver residing in the sinusoids (Surewaard et al., 2016). Importantly, no sequestration takes place by sinusoidal endothelial cells, hepatocytes or other liver cells. One explanation might be that hepatocytes have no direct access to the bloodstream and endothelial cells lack CRIg, the complement receptor required to trap *Staphylococcus aureus* under shear conditions (Helmy et al., 2006). SD-IVM was essential in identifying that CRIg directly binds to lipoteichoic acid—a cell wall component of gram-positive bacteria—independent of complement proteins or antibodies (Zeng et al., 2016).

Modern SDMs are not only able to image a large field of view e.g., to follow the fate of dozens of Kupffer cells; Using high magnification objectives allows to zoom into single cells and observe the processes unfold once bacteria have been phagocytosed. While the vast majority is destroyed by means of reactive oxygen species, a small fraction of staphylococci is able to survive and proliferate inside Kupffer cells after they have been phagocytosed (Surewaard et al., 2016)—hidden from the surveillance program of the innate immune system.

SD-IVM further helped to demonstrate that during staphylococcal infection, when bacteria are caught, platelets start to adhere to Kupffer cells, and help clearing bacteria through their von Willebrand factor (vWF) and fibrinogen receptors GPIb and integrin αIIbβ3, respectively (Wong et al., 2013). During sepsis, platelet aggregates can block blood vessels (van der Poll et al., 2017). Severity of *S. aureus* sepsis correlates with expression levels of its virulence factor α-toxin (Jenkins et al., 2015). A recent study using SD-IVM of the mouse liver and 2-photon IVM of the mouse kidney visualized the events following intravascular α-toxin injection and observed rapid platelet aggregation formation in the liver sinusoids and kidney glomeruli causing organ damage (Surewaard et al., 2018). Intravital microscopy therefore helped to unravel the Janus face of platelets in *S. aureus* bacteremia: On the one hand they assist Kupffer cells in clearing bacteria, on the other hand *S. aureus* α-toxin causes excessive platelet aggregation leading to organ dysfunction.

Solid organ transplantation offers a new lease on life to thousands of patients worldwide every year. To prevent graft rejection, patients have to be on lifelong immunosuppression after transplantation, which increases their risk of infection. In fact, infections are a leading cause of morbidity and mortality in the first year post-transplantation (Fishman and Issa, 2010). *S. aureus*, especially methicillin-resistant *S. aureus* (MRSA) is a serious threat to liver transplant recipients. Studies show that over 20% of patients develop MRSA infections with a mortality rate of 86% from MRSA pneumonia and 6% from catheter-related bacteremia (Singh et al., 2000). This was confirmed in a recent retrospective analysis of 2,700 transplant recipients that observed significantly increased likelihood of *S. aureus* infection in patients taking tacrolimus, a calcineurin inhibitor that is used to prevent graft rejection. In a mouse model of MRSA-induced sepsis using SD-IVM the authors observed that bacteria were more likely to disseminate and kill the host in tacrolimus-treated mice. Intravital imaging helped to uncover the underlying mechanism: reduced capacity of Kupffer cells to capture, phagocyte and destroy the bacteria (Deppermann et al., 2020).

Women show significantly lower disease severity and mortality from sepsis caused by Gram-negative bacteria such as *Escherichia coli* (Klein and Flanagan, 2016) suggesting they might be better at clearing bacteria from the bloodstream. Indeed, a sex difference in the capture of blood-borne bacteria by Kupffer cells could be shown using SD-IVM (Zeng et al., 2018). The female advantage was based on pre-existing antibodies against certain oligosaccharides in the bacterial lipopolysaccharide which were transferrable to pubs to provide protection during infancy. The fact that this antibody could also be found in humans demonstrates how useful intravital microscopy can be for preclinical studies which then also translate into the clinic.

### Visualizing Fast Platelet-Kupffer Cell Interactions Using SD-IVM

Platelets are small, anucleated cell fragments derived from bone marrow megakaryocytes that patrol the vasculature to maintain hemostasis. The human body produces 100 billion platelets every single day that circulate in the bloodstream for several days (Quach et al., 2018). At the end of their lifespan, platelets are cleared in the liver by a process that is incompletely understood (Aster, 1969). Previously, it had been suggested that platelets become desialylated as they circulate, that old platelets are phagocytosed by hepatocytes through their Ashwell-Morell receptor causing thrombopoietin production which then drives platelet production (Grozovsky et al., 2015). A recent study applied intravital microscopy to investigate the specific contributions of endothelial cells, hepatocytes and Kupffer cells in the clearing of aged, desialylated platelets. Using SD-IVM they found frequent interactions of platelets with Kupffer cells under steady-state conditions. The vast majority of interactions was short-lived, however, there was a small population that formed long-term interactions, which is in-line with previous studies describing continuous “touch-and-go” activity of platelets in the sinusoids of the liver (Wong et al., 2013). Using 3D reconstructions of z-stacks recorded of the liver, they found that indeed a small percentage of platelets is constantly being taken up by Kupffer cells. Upon desialylation, the vast majority of platelets rapidly bound to and was phagocytosed by Kupffer cells, with only a few binding to endothelial cells and basically none to hepatocytes. This unambiguously showed for the first time that Kupffer cells play an important role in the clearance of aged, desialylated platelets (Deppermann et al., accepted).

### Visualizing Neutrophil Extracellular Trap Formation

Neutrophil extracellular traps (NETs) are DNA filaments released from activated neutrophils coated with histones, proteases, and other granular proteins that entangle bacteria to support pathogen elimination. NET formation also takes place during sterile inflammation, e.g., in autoimmunity, coagulation and cancer (Jorch and Kubes, 2017) an can lead to organ damage (Jimenez-Alcazar et al., 2017). SD-IVM and resonance-scanning confocal intravital microscopy were used in combination with a mouse model of sepsis to show significant platelet aggregation, thrombin activation and fibrin clot formation downstream or within NETs *in vivo*. Thrombin generation was visualized using an internally quenched Förster resonance energy transfer (FRET) substrate. Active thrombin cleaves the FRET substrate resulting in the release of a fluorescent green dye. NET degradation *via* DNase infusion significantly reduced thrombin generation and platelet aggregation (McDonald et al., 2017).

Neutrophils are an essential part of the innate immunity and provide a first line of dense against invading bacteria and other pathogens. However, they are also recruited to sterile injury through tissue damage signals and this has been elegantly demonstrated in a study using a mouse model of focal hepatic necrosis and SD-IVM. Mice expressing enhanced green fluorescent protein under the control of the lysozyme M promoter (LysM-eGFP) were used to visualize the rapid “sprint” of neutrophils toward the injury site (McDonald et al., 2010). Besides ATP released from the injury, platelets surrounding the site were necessary for neutrophil recruitment and subsequent repair (Slaba et al., 2015).

### Immune Cells and Bacteria in the Peritoneal Cavity

The peritoneum harbors a large number of immune cells including B cells, mast cells, and different types of macrophages but under steady-state conditions no neutrophils. These peritoneal macrophages have been often used for *in vitro* experiments as they are easy to isolate. The number of studies that actually investigate the role of these cells in their natural environment *in vivo*, however, is limited. A phenomenon known since a long time is the “macrophage disappearance reaction” (Nelson, 1963). After injection of inflammatory stimuli like lipopolysaccharide or zymosan the macrophages become irretrievable through peritoneal lavage. Recently, two studies used different microscopy approaches that helped to understand the disappearance reaction. One study investigated the dissemination of *S. aureus* after it escaped from the liver Kupffer cells. Surprisingly, they discovered that the mesothelium of the liver ruptures and bacteria disseminate into the peritoneal cavity. There, *S. aureus* was first phagocytosed by large peritoneal macrophages (LPMs) thereby “hiding” the bacteria from detection by neutrophils. The LPMs were not able to kill *S. aureus* and in fact got overgrown and killed themselves from the bacteria within. At that point, neutrophils arrived in the peritoneal cavity. LPMs, which have been infected *in vivo* by bacteria that previously escaped KCs, were harvested to image them in culture *ex vivo* with common fluorescence microscopy. This rather straightforward-imaging technique nicely demonstrated that almost all LPMs got overgrown and killed by *S. aureus* while the neutrophils that infiltrated the peritoneal cavity at later time points were able to kill the bacteria (Jorch et al., 2019). The second recent paper, which directly investigated the macrophage disappearance reaction, used for the first time 2-photon IVM to visualize the inside of the closed peritoneal cavity. This imaging revealed that the macrophages are free floating and non-adherent under homeostasis but became adherent after i.p. injection of zymosan or *E. coli*. Coagulation factor V expression on the peritoneal macrophages was needed to form clots of macrophages and bacteria, which in turn was necessary to control bacterial expansion (Zhang et al., 2019). While imaging the closed peritoneal cavity could benefit other studies than peritonitis e.g., to investigate the immune- and cancer cell interactions in peritoneal metastatic disease which often arise from primary colorectal and ovarian carcinoma, it still has its limitations regarding depth and areas that can be observed. The macrophage-bacteria clots were isolated from the cavity and imaged *ex vivo* (Zhang et al., 2019). This gives two possible explanations for the macrophage disappearance reaction: Either the macrophages die during systemic infections or they become adherent and cannot be isolated anymore in the peritonitis model. It would be interesting to use intravital imaging to find out if in the systemic model clots are also formed and the macrophages become adherent, as maybe both mechanisms go hand in hand and the macrophages die after they formed clots.

### Imaging Macrophages in Organs With Natural Motions

Cavity macrophages do not only exist in the peritoneum, they also appear in other body cavities like the lung or the heart. Both organs are not trivial to use for intravital imaging because of their inherent motion. After myocardial infarction, macrophages play a central role in functional recovery of the heart. Until recently it was believed that recruited macrophages are exclusively monocyte-derived and induce a proinflammatory response (Heidt et al., 2014) while resident macrophages could proliferate locally and contribute to healing and non-healing responses (Epelman et al., 2014; Dick et al., 2019). Imaging the cleared infarcted heart in combination with reporter mice has revealed a new population of pericardial cavity macrophages that relocate from the pericardial cavity to the ischemic heart and help to prevent post-injury cardiac fibrosis (Deniset et al., 2019). Recently, using cleared heart tissue and lightsheet microscopy, cardiac-resident macrophages were found to contribute to mitochondrial homeostasis in the heart and prevent ventricular dysfunction (Nicolas-Avila et al., 2020). Regarding intravital imaging of the heart, there have been a few different approaches including high frame rate imaging, micro-endoscopy or mechanical stabilization but all of these studies have been limited to the epicardial layer because of limitations of imaging depth. However, micro-endoscopy provided evidence that following acute myocardial infarction, monocytes are first recruited from the vascular reservoir and later from the spleen (Jung et al., 2013). More details about the current standard of intravital imaging of the heart are reviewed and summarized in Allan-Rahill et al. (2020).

For the lung the most widely used imaging technique involves placing an imaging window its surface while applying gentle suction (Looney et al., 2011). Using this technique in combination with SD-IVM, Neupane and colleagues could now demonstrate that, contrary to previous assumptions, alveolar macrophages are not sessile and are able to crawl from one alveolus to another through pores. Inhaled bacteria causes chemotaxis and phagocytosis of the intruders and blocking this process resulted in inflammation including neutrophil recruitment (Neupane et al., 2020). While of disadvantage in the peritoneal cavity, in the lung, where the alveoli are in contact with non-sterile air under homeostasis, it makes sense that inhaled bacteria are phagocytosed by macrophages without the recruitment of neutrophils that could cause further tissue damage.

### Imaging the Kidney

Examining the kidney with intravital microscopy is challenging because of the high tissue density and therefore limited penetration depth. As demonstrated in Figure 1, imaging the cortex region is possible using SD-IVM. Using this approach, Sedin and colleagues could recently demonstrate, that after light-induced sterile tissue injury and after microinfusion of uropathogenic *E. coli* into a single nephron, neutrophils rapidly accumulated at the injury or infection site while the number of renal mononuclear phagocytes was not increased (Sedin et al., 2019). However, this study does not reflect the usual transition of bacteria from the bladder to the kidneys *via* the ureter. To detect ascending bacteria with intravital approaches at least 2-photon IVM is necessary. An even more elegant approach would be the usage of an abdominal imaging window on the kidney. This approach allows to observe the same kidney over time and was used to study renal epithelial cells and podocytes (Schiessl et al., 2020) but not yet to investigate immune cells in pyelonephritis or other pathophysiologic conditions like acute kidney injury or glomerulonephritis that progress over time. Nevertheless, the limited tissue penetration will most likely not allow to study the medulla region of the kidneys. Here, clearing techniques become relevant to at least be able to investigate the specimen in a spatial manner, as the organs can first be screened with lightsheet microscopy and afterwards, for better resolution, the region of interest of the cleared organ can be analyzed with 2-photon microscopy. As cleared organs can be imaged from all sides and penetration of light is much deeper, it is usually possible to visualize the region of interest. Lightsheet microscopy helped to determine the total number of glomeruli and their capillary tufts size in mice, which allowed to quantify the average creatinine clearance rate per glomerulus under steady-state and experimental nephrotoxic nephritis, where first the average creatinine clearance rate per glomerulus decreases followed by the total number of glomeruli (Klingberg et al., 2016).

## Conclusion and Perspective

Intravital imaging has provided new insights into immunologic processes in a large number of organs and diseases. Combining mouse models with IVM has helped us understand (patho)physiologic processes and to unravel the underlying mechanisms. With IVM and relevant mouse models becoming more widely available, we expect that it will help to solve research questions in many different fields.

## Author Contributions

SJ and CD conceptualization. SJ and CD writing—original draft. SJ and CD writing—review and editing. Both authors contributed to the article and approved the submitted version.

## Conflict of Interest

The authors declare that the research was conducted in the absence of any commercial or financial relationships that could be construed as a potential conflict of interest.
